# Anti-TNF Therapy in Crohn’s Disease

**DOI:** 10.3390/ijms19082244

**Published:** 2018-07-31

**Authors:** Samuel O. Adegbola, Kapil Sahnan, Janindra Warusavitarne, Ailsa Hart, Philip Tozer

**Affiliations:** 1 Robin Phillips Fistula Research Unit, St Mark’s Hospital and Academic Institute, Harrow HA1 3UK, UK; ks303@doctors.org.uk (K.S.); colorectal@btinternet.com (J.W.); ailsa.hart@nhs.net (A.H.); philtozer@doctors.net.uk (P.T.); 2 Department of Surgery and Cancer, Imperial College, London, South Kensington Campus, London SW7 2AZ, UK

**Keywords:** anti-TNF, Crohn’s disease, medical treatment

## Abstract

Crohn’s disease (CD) accounts for a variety of clinical manifestations or phenotypes that stem from chronic inflammation in the gastrointestinal tract. Its worldwide incidence is increasing including younger or childhood-onset of disease. The natural history of Crohn’s disease is characterized by a remitting and relapsing course that progresses to complications and surgery in most patients. The goals of treatment are to achieve clinical and endoscopic remission, to avoid disease progression and minimise surgical resections. Medical treatment usually features antibiotics, corticosteroids, immunomodulators (thiopurines, methotrexate). Anti-TNF (tumour necrosis factor) therapy was approved for use in Crohn’s disease in 1998, and has changed the paradigm of treatment, leading to improved rates of response and remission in patients. There are significant considerations that need to be borne in mind, when treating patients including immunogenicity, safety profile and duration of treatment.

## 1. Introduction

Crohn’s disease (CD) is a chronic inflammatory condition that can affect any part of the gastrointestinal (GI) tract. It is encompassed in the term “inflammatory bowel disease (IBD)”, which includes ulcerative colitis (UC) and CD and these are characterised by chronic, uncontrolled and relapsing inflammation of the GI tract. The incidence and prevalence of IBD is increasing with the highest annual incidence of CD being in North America (20.2 per 100,000 person-years) [1]. CD is regarded as a broad diagnosis with various phenotypes of immune mediated inflammatory disease of the GI tract, with T cell mediated tissue destruction [2,3]. It is associated with elevated pro-inflammatory cytokines, tumour necrosis factor (TNF or TNF-α) as well as interferon gamma and interleukin 12 [3,4]. The phenotypic manifestations of CD are relevant clinically, due to differences in disease behaviour and treatment options between these phenotypes, defined Montreal Classification [5]. The clinical features of CD vary according to the disease phenotype (inflammatory/stricturing/fistulising) but commonly include chronic diarrhoea, abdominal pain and weight loss. The course of CD is typified by periods of relapse and remission with recurrent cycles of inflammation leading to development of complications such as strictures and intestinal fistulas. Perianal Crohn’s disease represents a severe disease phenotype that affects a third of patients with CD [6]. It encompasses a range of manifestations that include perianal fistulas, anal stenosis, skin tags, fissures and ulceration, imposing considerable burden on patients with CD. The therapeutic goal in CD is to induce and maintain remission, heal the mucosa and optimize quality of life for the patient [7]. Consensus guidelines recommend “step-up” treatment with initial trials of immunosuppressive treatment (corticosteroid and steroid sparing immunomodulators) prior to starting anti-TNF agents [8,9], although there is increasing evidence advocating for early institution of anti-TNF treatment [10], the “top-down” approach, especially in severe disease phenotypes [11]. In this review, we focus on the use of anti-TNF therapy in Crohn’s disease, its mechanism of action, role and future directions in therapeutic use.

## 2. Biology of TNF

Human TNF is a member of a large family of proteins and receptors that are involved in immune regulation [12]. The secreted form of TNF is a 17 kD, non-glycosylated protein, which is cleaved from its cell-surface bound precursor by TNF α converting enzyme (TACE). Both the transmembrane precursor and the soluble TNF have biological activity, with soluble/secreted TNF acting at sites remote from TNF producing cells, whereas the transmembrane form acts as a receptor via cell-to-cell contact, transmitting signals to targets when cells are directly in contact with TNF receptor expressing cells. This form of transmission of biological activity is known as “outside-to-inside signal” or ‘‘reverse signal” [13,14,15]. TNF is mainly produced by monocytes, macrophages and T lymphocytes, but also by mast cells, granulocytes, fibroblasts and several other cell types [12]. Various stimuli result in its release and these include bacteria, viruses, immune complexes, super-antigens, tumour cells, radiation, and stress [16]. TNF is a highly pro-inflammatory cytokine that is involved in the induction of fever, insulin resistance, bone resorption, anaemia, the activation of granulocytes/T cells, and in sepsis. TNF reacts with two distinct receptors (TNF receptor 1–expressed on all nucleated cells; and TNF receptor 2–preferentially expressed on endothelial and haematopoietic cells [15,17]) through which they exert their biologic effect. These effects are pro-inflammatory in nature and occur through increased production of proinflammatory cytokines, including interleukin (IL)-1β and IL-6, expression of adhesion molecules, proliferation of fibroblasts and procoagulant factors, as well as initiation of acute-phase responses, and inhibition of apoptosis of inflammatory cells [18,19]. It is involved in key processes in inflammation including the activation of coagulation and fibrinolytic responses, promoting the necessary neutrophil-endothelial adhesion necessary for recruitment to sites of inflammation [20,21,22], and promoting granulomatous inflammation through its role in recruitment of component cells (T lymphocytes, monocytes and macrophages) [16,23,24]. It activates leukocytes and induces acute-phase reactants and metalloproteinases and also inhibits apoptosis of inflammatory cells [25]. 

## 3. Role of TNF in the Aetiopathogenesis of CD

The underlying cause of CD is not fully understood; however, the aetiopathogenesis is thought to involve an interplay between environmental triggers, dysbiosis, aberrant immune responses/immunoregulation and genetic susceptibility [3]. CD results in mucosal injury and inflammation, whereby the epithelial barrier is breached as a primary or secondary event, and the luminal microflora stimulates a proinflammatory immune response. Mucosal injury and damage are associated with dysbiosis, which potentially perpetuates the inflammatory cascade [26]. CD is associated with a T-cell mediated response, and the hallmark of pathogenesis is transmural inflammation, which is facilitated by increased proinflammatory cytokines, interferon gamma and interleukin 12 [3,4] as well as TNF. Increased secretion of TNF from lamina propria mononuclear cells has been found in the intestinal mucosa and TNF positive cells have been found deeper in the lamina propria and in the submucosa [27,28]. Studies have demonstrated that TNF is increased in both the stool of patients with active CD compared to controls (i.e., those with inactive disease or absence of CD), although serum concentrations have been less distinctive [29,30,31]. It is an early potent pro-inflammatory cytokine in the inflammatory process underlying CD [27,32] and has been demonstrated, in vitro, to be involved in the pathological processes (including neutrophil accumulation, granuloma formation, increased epithelial permeability) [33,34,35] seen in CD. 

## 4. Mechanism of Action of Anti-TNF Therapy in CD

Antibody neutralization studies implicated a significant role for TNF in the pathogenesis of Crohn’s disease [3]. These were initially done in animal models, before the first administration of an anti-TNF-α antibody cA2 (which later became infliximab, IFX) in a Crohn’s patient, followed by the first case series [36,37]. The results spurred on multicentre studies and led to the approval of anti-TNF agents in the treatment of CD by the Food and Drug Administration in 1998. Anti-TNF antibodies are thought to have multiple mechanisms of action including neutralization of TNF-α, reverse signaling, apoptosis, and cytotoxicity [38] and have a predilection and efficiency for distribution into inflamed tissue [15]. They are able to deplete overexpression of TNF-α, by binding soluble and transmembrane TNF-α and inhibiting binding to its receptors, resulting in blockage of proinflammatory signals or molecules that are upregulated by TNF-α. Anti-TNF treatment has also been shown in vitro to induce cytokine suppression via reverse signaling [13,39]. This interesting phenomenon occurs when the cell-surface bound precursor to TNF binds to anti-TNF and acts as a ligand and triggers cell activation, cytokine suppression or apoptosis of the cell bearing the cell-surface bound precursor [13,39,40]. It is thought that this is done via exhaustion of common signaling products during simultaneous endotoxin/lipopolysaccharide signaling pathway activation [38]. Anti-TNF also induces apoptosis of activated lamina propria T lymphocytes [41], countering a proposed pathological mechanism in CD, where mucosal T cell proliferation exceeds T cell apoptosis [42]. Anti-TNF therapies with an Fc region (i.e., infliximab and adalimumab but not certolizumab) are also able to induce antibody-dependent cell mediated cytotoxicity and complement-dependent cytotoxicity [15].

## 5. Types of Anti-TNF Treatment and Efficacy in CD

### 5.1. Infliximab

Infliximab was the first biological response modifier to be used in the treatment of inflammatory bowel disease [43] and is a genetically engineered chimeric (mouse/human) immunoglobulin (Ig)G1 anti human tumour necrosis factor agent. It has the ability to fix complement and lyse cells expressing membrane-bound TNF-alpha and induce of downregulation of the inflammatory mechanisms in the entire mucosal layer [44,45]. It is administered intravenously, typically on a maintenance schedule of every eight weeks after an initial three-dose induction. Two landmark randomised controlled trials, the ACCENT I and II studies [46,47] (A Crohn’s disease Clinical study Evaluating infliximab in a New long-term Treatment regimen) evaluated the efficacy of infliximab in patients with luminal as well as fistulising phenotypes of CD (see Table 1). These trials demonstrated the efficacy and safety of induction and maintenance therapy for moderate-to-severe CD as assessed by a disease activity grading system (CDAI—Crohn’s Disease Activity Index). In luminal disease, an increased likelihood for short and longer term remission, as well as discontinuation of corticosteroids (which would otherwise have been required to dampen the immune response), was found [46]. Subsequent studies have demonstrated that Infliximab treatment not only results in a positive clinical response, but also in a significant endoscopic improvement and histological examination confirmed that a complete reduction in the inflammation infiltrate could only be seen in the patients treated with infliximab [44]. Outcomes in perianal fistulising disease (rectovaginal/perianal fistulas) included the closure of draining fistulas [48]. In the first placebo-controlled trial of 94 patients (mostly with perianal fistulae), closure of ≥50% of the fistulas (primary endpoint) occurred in 68% (compared with 26% in placebo, *p* = 0.002) and closure of all fistulas draining at baseline occurred in 55% (compared with 13% placebo, *p* = 0.001) of the 63 patients receiving infliximab at 0, 2, and 6 weeks [49]. Maintenance therapy resulted in around a third of patients remaining in remission at one year [47]. However, clinical remission does not always reflect true deep tissue healing, which has been demonstrated on imaging (magnetic resonance imaging/endoscopic ultrasound) [50].

### 5.2. Adalimumab

Adalimumab, a fully human antibody that also fixes complement and lyses cells expressing TNF-α, is administered subcutaneously (via auto-injector pen) every two weeks. Crucial studies that demonstrated evidence for Adalimumab use in CD included the CLASSIC-I trial (Clinical Assessment of adalimumab Safety and efficacy Studied as Induction therapy in Crohn’s disease) [52] (Table 1). CLASSIC-I demonstrated efficacy in induction of clinical response and remission for moderate to severe CD and a subsequent phase 3 study, i.e., CLASSIC II demonstrated maintenance of remission [53] in those with moderate to severe CD. The CHARM trial [54] (Crohn’s trial of the fully Human Antibody Adalimumab for Remission Maintenance) also demonstrated safety and efficacy of adalimumab for maintenance of clinical remission following successful induction therapy and in the healing of draining perianal fistulas [60]. The GAIN (Gauging Adalimumab efficacy in Infliximab Nonresponders) trial assessed adalimumab efficacy in the context of loss of response or intolerance (secondary failure) to infliximab therapy [55]. The authors reported a significantly higher induction of clinical remission at week 4 in the adalimumab-treated group vs. the placebo-treated group. A meta-analysis including the CLASSIC-I, GAIN and CHARM studies, representing over 700 included participants with moderate to severe CD revealed a lower likelihood of failure to induce remission on adalimumab vs. placebo at weeks four and twelve [Relative risk (RR) 0.85, 95% confidence interval (CI) 0.79–0.91 [61,62]. In these studies, the measure of efficacy used was the CDAI (Crohn’s disease activity index) scores, with a decrease of 100 points signifying response and a decrease of 150 points signifying remission. The nature of the CDAI scoring system has been described as incorporating subjective symptoms, however, and a subsequent study went further in assessing efficacy using a more objective outcome measure; defining disease burden in terms of presence or absence of mucosal healing [63], the Extend trial found patients with moderate to severely active CD who continued to receive adalimumab were more likely to achieve mucosal healing.

### 5.3. Certolizumab

Certolizumab pegol is a chimeric humanized antibody fragment against soluble (secreted) TNF and transmembrane TNF, which is attached to polyethylene glycol, PEG (“pegylated”). It differs from IFX and ADA in that it does not contain the crystallisable fragment (Fc) region of typical antibody. It also differs in function in that it does not induce apoptosis as one of its mechanisms of action, nor does it activate the complement pathway, or result in cell or antibody mediated cytotoxicity [64,65]. It is however thought to have a higher binding affinity for TNF than adalimumab or infliximab [65]. Certolizumab is administered subcutaneously and has a longer half-life (due to the PEG addition), with maintenance dosing every four weeks (as opposed to adalimumab’s two weeks) (Table 2). The PRECISE (Pegylated antibody fragment evaluation in Crohn’s disease safety and efficacy) 1 and 2 [57,59] studies evaluated induction and maintenance of remission with certolizumab in patients with moderate to severe CD (Table 1). The PRECiSE 1 (Pegylated antibody fRagment Evaluation in Crohn's disease: Safety and Efficacy) study [57] did not find any significant difference in remission (at six weeks) between certolizumab and placebo groups; however, response rates were significantly improved with certolizumab vs. placebo (35% vs. 27% *p* = 0.02). The PRECiSE 2 trial [59] reported a significantly higher response rate (62% vs. 34%, *p* < 0.001) and remission rate (48% vs. 29%, *p* < 0.001) with maintenance of certolizumab following positive response to induction therapy at 26 weeks, compared to placebo. Certolizumab has also been evaluated using health related quality of life (QoL) as an outcome measure, by assessing patients’ response to treatment using the Inflammatory Bowel Disease Questionnaire (IBDQ) [66]. Rutgeerts et al. [66] reported a significantly improved QoL in patients with certolizumab at all time points assessed compared with placebo. The PRECiSE 3 trial assessed long-term outcome in patients successfully maintained on certolizumab at 26 weeks and reported remission rates of 63% at 80 weeks [67]. This was not statistically significantly different from those in whom the drug was stopped at 26 weeks (placebo). A meta-analysis [61] of four trials assessing certolizumab in over 800 patients found no statistically significant difference between certolizumab and placebo in inducing remission of active luminal CD (RR = 0.95; 95% CI 0.90–1.01). Maintenance therapy with certolizumab has demonstrated efficacy in perianal fistula closure. In a subgroup analysis of the PRECiSE 2 trial, 58 patients with draining fistulas who responded to induction with certolizumab were randomized to certolizumab or placebo every four weeks, with rates of clinical remission (100% closure of fistulas at baseline) at week 26 significantly higher in patients treated with certolizumab as compared with placebo (36 versus 17 percent *p* = 0.038) [68]. The above definition of clinical remission (i.e., 100% closure of fistulas at baseline), was updated from the initial protocol definition of fistula closure (i.e., ≥50% closure at two consecutive post-baseline visits ≥3 weeks apart). Data analysis using the initial protocol definition of clinical remission resulted in no significant between the groups. As exemplified in the above example, multiple definitions of success (or remission) in fistula management have been reported and these often limit the ability to discern true long-term sustained fistula closure rates; better outcome measures have recently been proposed by consensus [69].

There are no trials comparing all three anti-TNF therapies, however there are suggestions that there are no significant differences in efficacy between infliximab and adalimumab [70]. They are both also thought to be superior to certolizumab in inducing remission [71,72,73]. In a pooled analysis of ten trials including over 2700 patients with Crohn’s disease, patients who were treated with any of the three different anti-TNF agents discussed above were less likely to fail to achieve remission compared with placebo (RR 0.87, 95% 0.80–0.94) [61]. 

The efficacy of anti-TNF medication has led to the introduction of biosimilars, as the patents of older anti-TNF agents have either expired or are close to expiration. Biosimilars are synthesized versions of existing biological drugs with no perceived difference in safety or efficacy. Several have been approved for treatment of CD in USA and Europe (with 19 products authorised at the end of 2015 [74]) and they are expected to gain a substantial portion of the market of biological therapy in the future [75]. They have the advantage of lower cost, reducing health-care spending and making them more accessible to a larger number of patients [74]. Biosimilar development requires selection of an appropriate reference biologic agent, understanding of the key molecular attributes of the reference product, development of a manufacturing process to match these attributes, and finally preclinical and clinical evaluation. This includes pharmacokinetic/pharmacodynamic studies, randomized controlled trials, etc. [76]. Preliminary data from real-life cohort studies across different countries that may support the bioequivalence of infliximab biosimilars in IBD, as well as in rheumatology and dermatology [74,77]. Gecse et al. found a significant decrease in the clinical activity index and C-reactive protein (CRP) in the whole study population (inclusive of CD and UC), and only four allergic reactions in subjects previously exposed to infliximab. Similar preliminary results come from the large prospective study in Norway (the NOR-SWITCH study) [78]. It has reported that switching to a biosimilar infliximab was noninferior to continued treatment with the reference product in terms of efficacy, safety, and immunogenicity. However, the trial wasn’t powered to demonstrate the noninferiority of the biosimilar in individual disease states, including CD and UC. The preliminary data suggest that little difference is anticipated from the use of biosimilars of infliximab compared to the originator. There is, however, contrasting data from an Irish cohort [74,77] that compared two groups of patients treated with infliximab originator or biosimilars, showing an increased surgery rate, less steroid-free remission rates, and less normalization of inflammatory marker CRP. Further appropriately designed observational studies and efficient pharmacovigilance programmes that improve biosimilars’ safety profile are warranted [79] to address implications of these new drugs and whether existing techniques of drug monitoring, efficacy and safety application. Furthermore, head-to-head trials to assess best treatment pathways [26], as well as close cooperation of regulatory authorities, scientific societies and the pharmaceutical industry would serve to improve knowledge and clinical practice guidelines to standardize the use of biosimilars [76,80].

## 6. Loss of Response to Anti-TNF Therapy

Despite the paradigm shift seen over the last two decades with anti-TNF treatment in Crohn’s disease, the response that an individual patient will have to a specific anti-TNF and dose is difficult to predict when compared with conventional (non-biologic) therapies. Up to 30% of patients do not respond to anti-TNF therapy (primary non-responders) and almost half of the patients who experience a benefit with these drugs will lose clinical benefits within the first year, requiring dose escalation or therapy change [55,81,82], termed “secondary loss of response”. Several studies have demonstrated benefits in empiric switching to a different anti-TNF agent following loss of response; however, short-term remission rates were modest [79,83,84].

The reasons why some patients do not respond or lose response after a successful course of therapy are not completely clear, but they are likely multifactorial and related to metabolism of the drug or to the development of antidrug antibodies [81]. This process is termed immunogenicity and results from exposure to anti-TNF, inducing cellular clonal expansion of lymphocytes that secrete specific antibodies that form immune complexes with anti-TNF agents. This often results in increased drug clearance via the reticuloendothelial system. Pre-treatment anti-drug antibodies have been reported in treatment-naïve patients, and levels were found to be higher in those who lost (primary and secondary) response when compared to responders [85]. Moreover, patients with secondary loss of response have been found to have higher anti-drug antibodies levels than those with primary loss of response. This process is not permanent, as anti-drug antibodies have been found to disappear within a year of discontinuing the anti-TNF treatment, and furthermore a subset of patients may spontaneously lose pre-existing anti-drug antibodies whilst on continuous anti-TNF treatment [85,86]. The latter makes the case for dose escalation following loss of response as well as consideration of a switch in anti-TNF agent following loss of response [82]. A systematic review of CD patients switching from infliximab to a second anti-TNF-α agent (i.e., adalimumab/certolizumab) revealed a clinical response rate of 63% and remission rate of 43% [87]. Concomitant therapy with immunomodulators (i.e., azathioprine or methotrexate) reduces the risk of development of anti-drug antibodies [51,88]. The SONIC group (Study of Biologic and Immunomodulator Naive Patients in Crohn’s Disease) reported on a randomised controlled trial with azathioprine monotherapy, infliximab monotherapy or combination therapy in azathioprine and infliximab naive CD patients [51]. Formation of anti-drug antibodies was significantly less with scheduled infliximab combination therapy (0.9%) compared with infliximab monotherapy (14.6%). Combination therapy is also associated with higher infliximab serum levels and although the mechanism for this is unclear, infliximab serum levels at week 4 after the first infusion have been shown to be predictive of anti-drug antibody formation [89]. Measurement of serum anti-TNF trough levels and anti-drug antibodies (see therapeutic drug monitoring) are thus helpful in guiding treatment. Other potential therapeutic strategies to reduce the risk of anti-drug antibodies include the use of scheduled administrations of anti-TNF agents instead of episodic/’on-demand’ treatment, which is associated with a higher rate of antibody formation, infusion reactions [90] and the risk of undergoing abdominal surgery) [89,91].

Other factors that may result in loss of response are potential differences in pharmacokinetic properties, including tissue penetration and mode of action of the different anti-TNF drugs. The need to develop biomarkers that can predict response to therapies will become increasingly important for personalised medicine decisions in the future. Once loss of response is confirmed, dose escalation/interval shortening and switching anti-TNF agents are recommended potential strategies [9].

## 7. Pharmacokinetics and Anti-TNF Monitoring in CD

The route of administration of anti-TNF influences its pharmcokinetics. Infliximab is administered intravenously, which allows for the usage of large volumes, rapid distribution, with peak serum concentrations shortly after infusion and low variability in bioavailability [92]. Adalimumab and certolizumab on the other hand, are subcutaneously injected, which improves the practicality and convenience in administration, but leads to higher variability in bioavailability (due to low injectable volumes), necessitating more frequent administrations. The subcutaneous route has been reported to be more likely to illicit an immunogenic response in part due to activation of dendritic cells present in skin [34].

Antibody distribution is mainly within the extracellular fluid and occurs via cellular movement from circulation to tissue, diffusion or receptor mediated endocytosis [73,93]. For subcutaneously injected drugs, distribution can be slower, as it occurs through the lymphatic drainage and paracellular movement leading to assimilation into the vasculature [92]. Metabolism and clearance of the antibodies are not fully understood, but is thought to involve various processes including degradation in lysosomes following immune complex (anti-TNF/trans-membrane TNF) formation and catabolism by phagocytic cells. The latter occurs via the reticuloendothelial system via proteolytic catabolism, which occurs at sites that are in rapid equilibrium with plasma [81]. Immunogenicity/development of anti-drug antibodies increases clearance rates and can contribute to a loss of response to the anti-TNF (see above). This varies between individuals and risk is increased with episodic or on demand anti-TNF treatment (see above—the Loss of response section). Furthermore, clearance rates can be affected by concomitant medication and disease state, with inflammatory tissue found to have significantly higher levels of TNF and anti-TNF, compared to matched uninflamed samples [92,94]. The serum concentrations of infliximab, adalimumab and certolizumab all have a linear relationship with administered dose and half-life is between 8–10 days for infliximab, 10–20 days for adalimumab, and 14 days for certolizumab. Serum drug concentration monitoring is used to ensure therapeutic dosing and avoid toxic concentrations (see below).

### Therapeutic Drug Monitoring

The principles of anti-TNF dosing regimen are to achieve steady-state range of serum or tissue drug concentrations that are adequate for the drug to neutralize surplus TNF, but not so high that it affects safety because of neutralization of homeostatic concentrations of TNF required for host defence. Conversely, tissue drug concentrations should not be so low as to impair efficacy as a result of suboptimal neutralization of TNF [12]. Within an individual patient, a linear relationship exists between anti-TNF dose and serum medication levels [95]. However, variability in the pharmacokinetics between patients makes it difficult to predict medication levels between patients. Measuring drug levels and determination of the presence of antidrug antibodies has the potential of identifying those who will benefit from dose escalation and those who will be best served by switching to an alternate drug within or outside the class, e.g., in the event of loss of response. Therapeutic drug monitoring thus has the potential to help physicians to improve and personalize the management of Crohn’s disease [7]. This could be done via serum level guiding dose escalation, or initiation of immunomodulators to maximise drug efficiency. This has been supported by studies assessing dosing based on symptoms/disease markers, versus dosing based on trough levels [95]. Patients with sub therapeutic trough levels were found to have significantly higher markers of inflammation (i.e., C-reactive protein) than those with optimal trough levels [95]. Analysis of patients from the ACCENT 1 study also demonstrated that those who failed to respond to therapy had lower serum infliximab concentration than those with a sustained response [46]. Early therapeutic drug monitoring (TDM) could be used to prevent loss of response rather than to reverse it (although further research is required to corroborate this [79]), as well as to optimize treatment with anti-TNF antibodies based on actual exposure to the drug rather than according to a standard dosing regimen. 

The use of TDM to optimise anti-TNF drug concentrations has a promising potential for dose optimisation in clinical practice, in view of the reported correlations between anti-TNF trough concentrations, anti-drug antibodies, and disease outcomes; however, studies in TDM are yet to demonstrate unequivocal benefit [96,97]. Two controlled trials, which investigated the clinical use of TDM based on drug concentration or symptoms, showed that trough-level-based dose intensification was not superior to dose intensification based on symptoms alone [26]. Concentration-based dosing was associated with fewer disease flares [96] but did not increase clinical, endoscopic or steroid-free remission in patients with active luminal CD [97]. The recent American Gastroenterological Association (AGA) guidelines in TDM [98] provide an overview and commentary on this evolving field and sets a framework for clinical management, albeit highlighting important gaps in current knowledge in this aspect of the care of individuals with IBD [99]. AGA guidelines specify target levels of anti-TNF drugs that are associated with clinical outcomes and also details issues related to the assessment of anti-drug antibody levels, for example, the inconsistency in results obtained with available assays. This raises the suggestion that anti-drug antibodies interpretation may need to be more regional and patient specific [99]. Based on the currently available evidence (often scanty), the suggested target trough concentrations are >5 mg/mL for infliximab, >7.5 mg/mL for adalimumab, and >20 mg/mL for certolizumab pegol. These are proposed as guides to decide whether escalation of therapy may be beneficial (if trough is below this threshold) compared with switching therapy (to be considered if trough is above this threshold) to achieve clinical response in patients who are experiencing secondary loss of response on maintenance therapy [100]. For asymptomatic patients with ongoing endoscopic activity or with perianal disease [101] who undergo reactive TDM, target trough concentrations may be higher, such that escalating index therapy may be a preferable option before switching therapies in these settings. It is also important to note that these guidelines are for IBD and are not uniform trough levels that need to be targeted for all patients regardless of clinical status or disease phenotype [98]. Further studies are required to determine exactly when trough levels should be measured, whether or not in combination with anti-drug antibodies, what the optimal trough level concentrations should be and if the dose should be adapted to this target (“treat-to-trough” approach) as well as whether or not these strategies improve quality of life, and cost-effectiveness [89]. Furthermore, the role of combination therapy (an immunomodulator in combination with the biologic agent) and the effect on TDM need to be elucidated.

## 8. Withdrawal of Anti-TNF Therapy

Clinicians and patients are often faced with the question on whether it is possible to stop anti-TNF therapy once disease remission has been achieved. However, despite all the studies that have now addressed this issue in IBD, no conclusive strategy has yet emerged [102]. Data is inconclusive due to varying reported rates of relapse on stopping anti-TNF, and conclusions are difficult to draw from the studies due to disease phenotype heterogeneity, with variable definitions of clinical remission and variable duration of remission before drug withdrawal. It is not known the ideal duration of remission prior to stopping as well as in which phenotypes this is most likely to be successful. There was also a lack of control groups. 

It is often empirically proposed not to routinely stop anti-TNF-α agents in IBD patients who respond, and especially in patients with disabling features of disease and/or at high-risk for relapse [103]. The STORI trial (Infliximab diSconTinuation in Crohn’s disease patients in stable Remission on combined therapy with Immunosuppressors) was the pivotal study boosting clinical research in this topic, being thereafter followed by many studies. This was the first prospective multicentre study specifically designed to assess the risk of relapse, and to identify predictors of relapse following anti-TNF maintenance therapy withdrawal [104]. Among the 115 CD patients with luminal disease that were enrolled (perianal CD was excluded), there was a 43.9% (±5.0%) rate of relapse over one year and a 52.2% (±5.2%) rate of relapse over two years after stopping IFX. Relapse occurred after a median of 16.4 months. Other retrospective and prospective cohorts have also sought to address the issue of treatment withdrawal [78]. In most of the studies on withdrawal, patients had the anti-TNF discontinued while they were in clinical remission (with variable definitions of clinical remission and variable duration of remission before drug withdrawal). Relapse rates among those studies range from 21 to 56% at 12 months and from 47 to 64% at 24 month [104,105,106,107,108,109]. Further studies are still required in order to answer the question on whether maintaining the anti-TNF as opposed to reducing/discontinuing the drug is superior to maintain remission; as well as to define routine strategy in the future for long-term management of CD patients and to define the optimal withdrawal strategy [102,103]. Studies assessing withdrawal in circumstances such as early disease or in the context of monotherapy (as opposed to in combination with immunomodulator agent), and a large European trial (Biocycle Project) seeks to answer some of these questions [102].

## 9. Adverse Effects of Anti-TNF Therapy in CD

Long-term therapeutic use of anti-TNF carries with it safety issues which include potential for development of skin lesions, immune reactions, peri-operative complications, infections, cancers and decreased fertility/adverse effects on pregnancy [79,110].

Infusion reactions relate to infliximab and can be categorized based on their timing, pathogenesis, and severity [111]. They are common within 1–2 h after an infusion and occur in about 20% of patients [25]. Symptoms include itching, flushing, breathlessness, chest pains, hypertension and headaches. Delayed reactions occur within 1–14 days following infusion and are rarer, occurring in about 2% of patients, usually in the form of headache, fever, fatigue, rash, myalgia and arthralgia [112,113]. Reactions have been associated with the presence of anti-drug antibodies [114,115], whereas concomitant immunomodulator use, and regular maintenance dosing have been shown to reduce the risk of infusion reactions by decreasing the incidence of anti-drug antibody formation [38,111]. Injection site reactions have also been reported [52] to occur following subcutaneous administration of anti-TNF therapy, (i.e., adalimumab/certolizumab) which can cause symptoms including burning sensation, pain, and pruritus.

Susceptibility to infection is a significant concern following instigation of treatment with anti-TNF. Anti-TNF treated patients are rendered immunocompromised through their treatment and this is often in the context of combination treatment with other immunosuppressant medication (e.g., corticosteroids, thiopurines, etc.). Infective complications vary from serious invasive bacterial/opportunistic infections to relatively milder cases (mild respiratory/urinary infections) [116]. Analysis from the CD TREAT registry (Crohn’s Therapy, Resource, Evaluation and Assessment Tool), which incorporates data on more than 3000 patients on anti-TNF therapy, revealed that infliximab-treated patients displayed a significantly increased risk of serious infections compared with the ‘other treatments-only’ group (hazard ratio = 1.45, *p* = 0.008). However, multivariate analysis, demonstrated no significant increased risk of infection (OR 0.99, 95% CI 0.64–1.53) due to anti-TNF therapy after controlling for factors such as disease duration, severity and concurrent corticosteroid and immunomodulator use [117]. In a report of adalimumab safety including six clinical trials, 1.8% of patients had opportunistic infections (most commonly oral candidiasis), and 5.8% had serious infections (most commonly abscess, gastrointestinal, pulmonary and viral infection) [118,119]. Anti-TNF therapy has also been implicated in the susceptibility to tuberculous infection, due to the role of TNF in the formation of granulomas. It is thought that suppression TNF-α prevents adequate sequestration of Mycobacterium tuberculosis [120], which in turn leads to an increased risk that anti-TNF therapy could cause reactivation of latent tuberculosis [38]. In view of the above, consensus guidelines advocated vaccination and safety screening, with screening for risk of opportunistic infections and ascertainment of immunisation status prior to starting anti-TNF treatment [116].

Anti-TNF therapy has been theoretically linked with a propensity for malignancy, due to the suppression of TNF, and a meta-analysis in 2006 reported a 3-fold increased risk of malignancy for patients with rheumatoid arthritis undergoing anti-TNF (infliximab/adalimumab) treatment [121]. However, this rate of malignancy is yet to be confirmed in Crohn’s disease treatment. An analysis of the FDA’s adverse event reporting system (AERS) database from 1968–2005 found a relatively strong signal for lymphoma, both in IBD and non-IBD patients, at the disproportionality analysis [122]. The risk of T-cell non-Hodgkin lymphoma was found to be increased in combination therapy use (95% confidence interval (CI) 4.98–354.09; *p* < 0.0001) and thiopurines alone (95% CI 8.32–945.38; *p* < 0.0001) but not with anti-TNF use alone (95% CI 0.13–10.61; *p* = 1.00) [119,123]. There are also data suggesting an increased risk of melanoma in patients on anti-TNF treatment for IBD, with a 1.5−2× increased risk compared to those not exposed [124], however this is also impacted by an increased risk of melanoma associated with IBD independent of the use of biologic therapy [125,126]. However, in the TREAT registry, the global incidence of neoplasia (malignant, benign, and unspecified) was similar between patients receiving infliximab and the other-treatments group (0.78 vs. 0.85 per 100 patients-years; RR = 0.90, CI 0.69–1.18, *p* = 0.46), with no differences seen for solid tumours, non-melanoma skin cancers, and lymphoma between the two groups [117]. In a meta-analysis of 21 studies enrolling 5356 CD patients, Peyrin–Biroulet and colleagues reported that anti-TNF therapy did not significantly increase the risk of death, malignancy, or serious infection when compared with placebo [127]. This has been corroborated in several other studies [128,129,130]. The evidence is mixed and this is unsurprising, as the risk of malignancy associated with treatment can be particularly difficult to evaluate in trials due to the rarity of cancer as an outcome and resultant paucity of data. Furthermore, the multifactorial aetiopathogenesis of malignancy, predisposition to cancer from the underlying disorder as well as unquantifiable risk from other medication can contribute to difficulty in identification of an appropriate control group. The 2016 European Crohn’s and Colitis organisation (ECCO) consensus statement concluded that there were currently insufficient data to suggest that anti-TNF agents alone increase the risk of lymphoproliferative disorders or solid tumour, and acknowledged the increased risk when used in combination with thiopurines, concluding that absolute rates of these malignancies remain low and risks and benefits of treatment should be discussed with the patient [9].

All three anti-TNF agents have been deemed safe in pregnancy, not least because of the increased risk of complications, preterm delivery and low birth weight conferred by active uncontrolled disease [89]. However, most of the available data are from animal and retrospective studies, with no teratogenic effects observed [119]. Infliximab and adalimumab cross the placenta in the beginning of the second trimester, whereas certolizumab does not as it lacks the Fc fragment required for active transport to the foetus [89]. Serum concentrations of infliximab may be detected up to six months after delivery, and may be predisposed to opportunistic infection and, as such, live vaccines are not recommended for children of patients on anti-TNF therapy in the first six months after birth [88,131].

## 10. Conclusions

In summary, anti-TNF introduction has led to major shifts in the therapeutic paradigm that evolved from slow symptomatic clinical remission towards rapid sustained and deep remission; short- and long-term clinical, and endoscopic endpoints can now be reached that were previously unachievable [89,92]. Anti-TNF treatment currently represents a central treatment modality in Crohn’s disease and can be improved by rapid dose escalation and the use of combination therapy [102]. However, two decades on from their introduction, questions still remain about their use including timing, dosing, monitoring and issues surrounding loss of response such as risk factors, biomarkers, mechanism and strategies for prevention and treatment [79]. These will undoubtedly form the basis of research in the future.

## Figures and Tables

**Table 1:** Summary of significant studies of anti-TNF in moderate to severe Crohn’s disease patients.

Study	Drug	Patient Groups	Response (Where Reported)	Remission
Targan et al. [4] 1997 Multicentre Double-blind placebo controlled trial	IFX	3 Treatment groups: Infliximab–5 or 10 or 20 mg/kg Placebo	At week 4: 5 mg/kg group 81% (22/27) 10 mg/kg group: 50% (14/28) 20 mg/kg group: 64% (18/28) Placebo: 17% (4/24)	At week 4: All dose treatment group–33% (27/83) Placebo group 4% (1/24)
ACCENT-I (Hanauer et al. [46]) 2002	IFX	3 treatment groups–same induction regimen (IFX 5 mg/kg at week 0) followed by: Group 1–Placebo at weeks 2 & 6 then every 8 weeks through to week 54 Group 2–IFX 5 mg/kg at weeks 2 & 6 then every 8 weeks through to week 54 Group 3–IFX 10 mg/kg at weeks 2 & 6 then every 8 weeks through to week 54	At week 2: For all participants receiving IFX 5 mg/Kg at week 0: 58% (335/573)	At week 30: (in those patients demonstrating clinical response at week 2) Group 1–21% (23/110) Group 2–39% (44/113) Group 3–45% (50/112)
ACCENT-II (Sands et al. [47]) 2004 Multicentre RCT	IFX	2 treatment groups–same induction regimen (IFX 5 mg/kg at week 0, 2, 6) followed by: Group 1–IFX 5 mg/kg every 8 weeks through to week 54 Group 2–Placebo every 8 weeks through to week 54	Median time to loss of response Group 1: >40 weeks Group 2: 14 weeks	At week 54–remission here refers to complete absence of draining fistulas Group 1–36% (50/138) Group 2–19% (27/144)
SONIC (Colombel et al. [51]) 2010 Multicentre RCT	IFX	3 treatment groups–all had IFX 5 mg/kg from weeks 8 through to week 50 In addition: Group 1: IFX 5 mg/kg at weeks 0, 2, 6 and azathioprine placebo daily Group 2: Placebo at weeks 0, 2, 6 and azathioprine 2.5 mg/kg daily Group 3: IFX 5 mg/kg at weeks 0, 2, 6 and azathioprine 2.5 mg/kg daily		*At week 26:* Group 1–44% (75/169) Group 2–30% (51/170) Group 3–57% (96/169) Mucosal healing in patients with ulcerations at baseline: Group 1 30% (28/93) Group 2 17% (18/109) Group 3 43.9% (47/107)
CLASSIC-I (Hanauer et al. [52]) Multicentre RCT 2006	ADA	4 treatment groups–initial dose (of ADA for groups 1–3 and placebo for group 4) at week 0, second dose at week 2, i.e., Group 1: ADA 160 mg/80 mg Group 2: ADA 80 mg/40 mg Group 3: ADA 40 mg/20 mg Group 4: Placebo/Placebo		At week 4: Group 1: 36% (27/76) Group 2: 24% (18/75) Group 3: 18% (13/74) Placebo: 12% (9/74)
CLASSIC-II (Sandborn et al. [53]) Multicentre RCT 2007	ADA	4 treatment groups– (1–3 were in remission at week 0, i.e., week 4 of CLASSIC-I, group 4 were not in clinical remission following treatment in CLASSIC-I) Group 1: ADA 40 mg fortnightly from 0 though to week 56 Group 2: ADA 40 mg weekly from 0 though to week 56 Group 3: Placebo through to week 56 Group 4: ADA 40 mg fortnightly through to week 56 (with allowance for decreased interval, i.e., weekly if continued non-response/flare)		At week 56: Group 1: 79% (15/19) Group 2: 83% (15/18) Group 3: 44% (8/18) Group 4: 46% (93/204)
CHARM (Colombel et al. [54]) 2007	ADA	3 treatment groups–all groups had ADA 80 mg at week 0, 40 mg at week 2 then: Group 1: 40 mg fortnightly through to week 56 Group 2: 40 mg weekly though to week 56 Group 3: Placebo through to week 56		At week 26: Group 1–40% (69/172) Group 2–47% (74/157) Placebo–17% (29/170) At week 56: Group 1 36% (62/172) Group 2: 41% (64/157) Placebo: 12% (20/170)
GAIN Sandborn et al. [55] Multicentre RCT 2007	ADA	2 treatment groups Group 1: 160 mg at week 0, 80 mg at week 2 Group 2: Placebo at weeks 0,2	At week 4: Group 1: 52% (82/159) Group 2: 34% (56/166)	At week 4: Group 1: 21% (34/159) Group 2: 7% (12/166)
Schreiber et al. [56] Multicentre RCT 2005	CZP	3 treatment groups–all with treatment at weeks 0, 4, and 8 weeks. However different drug dosing: Group 1: 400 mg CZP Group 2: 200 mg CZP Group 3: 100 mg CZP Group 4: Placebo	At week 12: Group 1: 44% (32/72) Group 2: 36.1% (26/72) Group 3: 36.4% (27/74) Placebo: 35.6% (26/73)	At week 12: Group 1: 26% (19/72) Group 2: 19% (14/72) Group 3: 27% (20/74) Placebo: 23% (17/73)
PRECISE I (Sandborn et al. [57]) Multicentre RCT 2007	CZP	2 treatment groups Group 1: 400 mg at week 0, 2, 4 then every 4 weeks through to week 26 Group 2: Placebo at week 0, 2, 4 then every 4 weeks through to week 26	At week 6: Group 1: 35% (115/327) Group 2: 27% (87/325) *At week 6 AND 26:* Group 1: 23% (75/325) Group 2: 16% (52/325)	At week 6: Group 1: 22% (71/329) Group 2: 17% (57/326) *At week 6 AND 26:* Group 1: 14% (47/327) Group 2: 10% (32/326)
Sandborn et al. [58] Multicentre RCT 2011	CZP	2 treatment groups: Group 1: 400 mg at 0, 2, 4 weeks Group 2: Placebo at 0, 2, 4 weeks		*At week 6:* Group 1: 32% (68/215) Group 2: 25% (53/209)
PRECISE-II (Schreiber et al. [59]) 2007 RCT	CZP	2 treatment groups: all received 400 mg at 0, 2, 4 weeks then following assessment of week 6 response: Group 1: 400 mgs at week 8, 12, 16, 20, 24 Group 2: Placebo at week 8, 12, 16, 20, 24	Maintenance of response at week 26: Group 1: 63% (135/215) Group 2: 36% (76/210)	At week 26 (i.e., remission data in those who demonstrated response at week 6): Group 1: 48% (103/215) Group 2: 29% (61/210)

**Table 2:** Demonstrates the three available anti-TNF therapies in Crohn’s disease (CD).

Anti-TNF	Dosing for Induction and Maintenance	Route	Properties	Indications
**Infliximab** **(Remicade)**	**Induction** 5 mg at weeks 0, 2 and 6 **Maintenance** 5 mg (or 10 mg/kg) every 8 weeks	IV	Chimeric monoclonal antibody	Induction and maintenance of remission
**Adalimumab** **(Humira)**	**Induction** 160 mg (or 80 mg) week 0, 80 mg (or 40 mg) week 2 **Maintenance** 40 mg every other week or weekly	SC	Humanized monoclonal antibody	Induction and maintenance of remission
**Certolizumab** **(Cimzio)**	**Induction** 400 mg at weeks 0, 2 and 4 **Maintenance** 400 mg every 4 weeks	SC	PEG-conjugated Fab fragment of recombinant humanised monoclonal antibody	Induction and maintenance of remission

IV—intravenous; SC—subcutaneous, TNF—tumour necrosis factor, PEG—polyethylene glycol, Fab—humanized antibody fragment.
